# Click chemistry functionalization of self‐assembling peptide hydrogels

**DOI:** 10.1002/jbm.a.37460

**Published:** 2022-10-10

**Authors:** Joe T. Sharick, Angelina J. Atieh, Keith J. Gooch, Jennifer L. Leight

**Affiliations:** ^1^ Department of Biomedical Engineering The Ohio State University Columbus Ohio USA; ^2^ The Center for Cancer Engineering, The James Comprehensive Cancer Center The Ohio State University Columbus Ohio USA; ^3^ Davis Heart & Lung Research Institute The Ohio State University Columbus Ohio USA

**Keywords:** click chemistry, extracellular matrix, hydrogel, matrix metalloproteinase, self‐assembling peptide

## Abstract

Self‐assembling peptide (SAP) hydrogels provide a fibrous microenvironment to cells while also giving users control of biochemical and mechanical cues. Previously, biochemical cues were introduced by physically mixing them with SAPs prior to hydrogel assembly, or by incorporating them into the SAP sequence during peptide synthesis, which limited flexibility and increased costs. To circumvent these limitations, we developed “Click SAPs,” a novel formulation that can be easily functionalized via click chemistry thiol‐ene reaction. Due to its high cytocompatibility, the thiol‐ene click reaction is currently used to crosslink and functionalize other types of polymeric hydrogels. In this study, we developed a click chemistry compatible SAP platform by addition of a modified lysine (lysine‐alloc) to the SAP sequence, enabling effective coupling of thiol‐containing molecules to the SAP hydrogel network. We demonstrate the flexibility of this approach by incorporating a fluorescent dye, a cellular adhesion peptide, and a matrix metalloproteinase‐sensitive biosensor using the thiol‐ene reaction in 3D Click SAPs. Using atomic force microscopy, we demonstrate that Click SAPs retain the ability to self‐assemble into fibers, similar to previous systems. Additionally, a range of physiologically relevant stiffnesses can be achieved by adjusting SAP concentration. Encapsulated cells maintain high viability in Click SAPs and can interact with adhesion peptides and a matrix metalloproteinase biosensor, demonstrating that incorporated molecules retain their biological activity. The Click SAP platform supports easier functionalization with a wider array of bioactive molecules and enables new investigations with temporal and spatial control of the cellular microenvironment.

## 1 INTRODUCTION

Hydrogels, or hydrophilic polymeric networks, are a class of well‐established biomaterials that have been engineered to provide cells with a physiologically relevant 3D microenvironment.^1^ Due to their dimensionality, biochemical tunability, and tissue‐like mechanical properties, hydrogel systems can mimic in vivo extracellular matrix (ECM) in ways that 2D tissue culture plastic (TCP) cannot. The most commonly used hydrogel systems are derived from natural ECM proteins (e.g., matrigel, hyaluronic acid, collagen), which are biocompatible, relatively easy to use, and provide cells with biochemical cues that promote viability and proliferation. However, naturally derived ECMs often suffer from high variability, limited tunability of individual ECM variables, and ill‐defined biochemical compositions. Synthetic hydrogels have also been developed (e.g., polyethylene glycol [PEG]), which enable independent tuning of biochemical and mechanical properties, however, these synthetic systems lack the fibrous architecture of native collagen‐rich ECM. While some synthetic hydrogel systems have been designed to contain fibrillar structures,^2^, ^3^, ^4^ they remain overall nanoporous, and cells must degrade the matrix with proteases in order to migrate. Fibrous architecture is a critical regulator of cell‐matrix interactions, affecting cell adhesion, migration, proliferation, and drug response in vivo.^5^, ^6^ For example, tissues such as tendon and ligament, which are primarily exposed to unidirectional loading, have collagen fibers aligned parallel to the direction of the load,^7^ while tissues that are exposed to mechanical loading in multiple directions (e.g., the skin) have less aligned fibers.^8^ Additionally, in some cancers, increased ECM alignment is a known negative prognostic indicator.^9^, ^10^ In vitro studies show that fiber alignment proceeds and facilitates directional cell migration.^11^, ^12^, ^13^ In silico studies suggest that a fibrous microarchitecture greatly enhances force transmission and may allow mechanically mediated signaling between cells.^6^, ^14^, ^15^ A hydrogel model system that combines a fibrous architecture with independently tunable properties would enable new, more physiologically relevant studies of fundamental cell biology and drug efficacy.

Self‐assembling peptides (SAP) are short amino acid sequences (<40 amino acids) which form hydrogels with a fibrous structure similar to that of collagen I,^16^ and enable independent tuning of stiffness and cell‐binding site density.^17^, ^18^ These models have been used to study many aspects of basic cancer research,^19^ including ECM remodeling,^20^ metastasis,^21^, ^22^ drug sensitivity,^23^, ^24^, ^25^, ^26^ tumor‐stromal interactions,^20^, ^24^ and cell dormancy.^27^ SAP have also been used to study stem cell differentiation,^17^, ^28^, ^29^ the formation of microvascular networks by endothelial cells,^30^, ^31^ and as a tissue engineering scaffold.^32^, ^33^, ^34^, ^35^, ^36^

SAP have been developed from a number of different amino acid sequences. We have previously developed a SAP gel system that provides independent control of the biochemical and mechanical properties while presenting cells with a fibrous microarchitecture. This system is based on the previously described peptide sequence KFE (acetyl‐FKFEFKFE‐CONH_2_).^30^ KFE contains alternating hydrophobic‐hydrophilic residues, which allows it to form two antiparallel β‐sheet ribbons. These ribbons stack to form a double helical morphology, which then form long fibrils.^37^, ^38^, ^39^ Since KFE does not support cell adhesion,^16^ it provides a “blank slate” on which other functionalities can be added.^17^, ^30^, ^40^ This contrasts with the most widely used SAP, RADA16, known commercially as Puramatrix. Cells are able to adhere to RADA16 without additional adhesion ligands, limiting the ability to control and vary adhesion ligand density and identity.^16^ By adding an integrin binding sequence (GRGDSP) to the base KFE, we created a new SAP, “KFE‐RGD” (acetyl‐GRGDSP‐GG‐FKFEFKFE‐CONH_2_), that supports cell adhesion. By mixing various amounts of KFE and KFE‐RGD prior to gelation, the stiffness and the integrin binding site density of the resulting gel could be independently controlled.^40^

Incorporation of biological signals into SAP has previously been achieved through mixing of freely suspended, unbound proteins into the SAP prior to assembly (e.g., laminin^31^), or by modifying SAP peptides with short amino acid sequences, such as in early work by Semino et al., who demonstrated that cells could interact with SAPs containing cell adhesion sites such as YIGSR, RYVVLPR, AND TAGSCLRKFSTM.^41^ Since then, SAP have been synthesized with many other short sequences, such as RGD,^30^, ^40^, ^42^ IKVAV,^42^, ^43^ PDSGR,^44^ MMP‐sensitive PVGLIG,^45^ and other functional motifs.^46^ Incorporation of moieties on the SAP itself presents challenges. First, any moiety being added is constrained by the limitations of solid phase peptide synthesis, namely, it must be a short peptide (<40 amino acids), and it must exhibit the necessary solubility characteristics. These moieties must already be modified with the correct chemical groups (i.e., protecting groups) to make them compatible with SPPS, greatly increasing their cost. Additionally, the position of the moiety within the SAP and SAP block length may need to be optimized to maintain sufficient gel assembly, which can require significant peptide synthesis time and cost.

Therefore, methods that are not constrained by peptide synthesis to covalently incorporate biological signals into SAP could greatly increase the utility of the SAP system and enable incorporation of a wider range of molecules. We reasoned that functionalization through a simple thiol‐ene click chemistry process, hereafter referred to as “Click SAP,” could provide an alternative method to functionalize SAP. Click chemistry reactions are efficient and cytocompatible,^47^ and have been used to functionalize many hydrogel systems.^48^, ^49^, ^50^, ^51^, ^52^, ^53^, ^54^, ^55^, ^56^ Thiol‐ene chemistry can be initiated by UV light, which allows for precise spatial and temporal control of covalent bonding between a thiol group and an “ene” group. This feature of the reaction has enabled a new array of models of the cell microenvironment with higher spatiotemporal complexity and flexibility than previously feasible.

SAPs have previously been shown to be compatible with click chemistry reactions. One class of SAPs, known as collagen mimetic peptides, has been successfully conjugated into PEG gels^2^, ^3^, ^4^ and into elastin like peptide nanoparticles.^57^ The thiol‐ene reaction was also previously used to modify SAP fibers with a biotin‐containing peptide, which were then labeled with streptavidin gold nanoparticles as a contrast agent.^58^ However, click chemistry has not been previously used to functionalize SAP hydrogels for cell culture applications. Here we use click chemistry to introduce functionality into the blank slate of the KFE SAP, demonstrate that the resulting Click SAP retains the desirable mechanical and micro‐architectural properties of the original SAP, and illustrate the flexibility and utility of the Click SAP by introducing three different classes of functionality: a fluorescent label, an integrin binding site, and an MMP‐sensitive biosensor.

## 2 MATERIALS AND METHODS

### 2.1 SAP preparation and thiol‐ene chemistry

This study used several previously developed SAPs sequences, including KFE‐8 (referred to here as KFE), the cell adhesive sequence KFE‐RGD, and non‐adhesive sequence KFE‐RDG. ^30^, ^40^ A new sequence, KFE‐alloc, was created by adding a lysine with an alloc group to the N‐terminus of KFE (Table 1). All peptides were purchased from Biomer Technology, reconstituted in deionized, sterile water at 10 mg/ml, sonicated in a water bath for 30 minutes, and stored at −70°C.

**TABLE 1:** Peptide sequences

Peptide name	Sequence	MW (g/mol)
KFE	(acetyl)‐FKFEFKFE‐CONH_2_	1162
KFE‐RGD	(acetyl)‐GRGDSP‐GG‐FKFEFKFE‐CONH_2_	1846
KFE‐RDG	(acetyl)‐GRDGSP‐GG‐FKFEFKFE‐CONH_2_	1846
KFE‐alloc	K‐(alloc)‐GG‐FKFEFKFE	1447

Solutions containing various mixtures of the above SAPs, along with the indicated concentrations of photoinitiator, thiolated molecules, sucrose, and/or cells were mixed in sterile, deionized ultrapure water. Either 45 μl or 75 μl was pipetted into 24‐well plate Thincert hanging cell culture inserts (Greiner bio‐one, cat#662641), resulting in theoretical pre‐swelling thicknesses of approximately 1.3 and 2.2 mm, respectively, calculated by dividing the volume of gel by the area of the cell culture insert membrane. Thinner gels were used for all cell encapsulations, fluorescent dye labeling, and MMP biosensor studies, while the thicker gels were used for rheological measurements and 2D cell spreading studies. To assemble gels, 800 μl of media or PBS were added to the 24‐well plate below each hanging insert, which allows ions to slowly diffuse through the semi‐permeable PET membrane into the SAP solution and initiate gel formation. After approximately 25 min, the gels achieved sufficient assembly for additional media or phosphate buffered saline (PBS) to be pipetted on top of the gels.

The photoinitiator lithium phenyl‐2,4,6‐trimethylbenzoylphosphinate (LAP) was synthesized as previously described.^48^ To initiate the thiol‐ene reaction, gels or pre‐gel solutions containing KFE‐alloc, LAP, and either TAMRA, RGD, or MMP biosensor were exposed to 365 nm UV light for 60 seconds (or other durations as indicated) at a measured intensity of 3.87 mW/cm^2^.

### 2.2 Characterization of clicked‐in TAMRA

A short spacer, 3,6‐dioxaoctanoic acid linker (Chem‐Impex #07310), was added to a cysteine amino acid using DIC‐mediated (Sigma #D125407) solid phase peptide synthesis.^50^, ^59^ To this peptide, a 5(6)‐carboxytetramethylrhodamine (TAMRA) (Sigma #8.51030) fluorescent group was covalently attached on resin to the N‐terminus with N,N′‐diisopropylethylamine (Sigma #D125806) and 6 equivalents of HATU (Sigma #445460) in N,N‐Dimethylformamide solvent (Sigma #227056), and reacted at room temperature for 2.5 h. Reaction completion was confirmed by a negative ninhydrin test. After peptide cleavage, the TAMRA product was purified by reverse phase HPLC and verified by MALDI TOF mass spectroscopy (M = 678.2 [M + H]).

SAP hydrogels with clicked‐in TAMRA dye were generated with a volume of 45 μl, containing 3.2 mM KFE, 0.54 mM KFE‐alloc, 2 mM LAP, and 0.1 mM TAMRA and gels were formed as before. TAMRA was not included at the stochiometric equivalent to KFE‐alloc due to its strong fluorescent signal. The click reaction was initiated using 60 s of UV light, and fluorescence was immediately quantified (0 h post‐UV measurement). Cell culture inserts containing the gels were rinsed throughout the experiment in PBS and rocked at room temperature, with a change of the PBS occurring before the 1 h post‐UV measurement. Fluorescence measurements of the TAMRA within the hydrogels were conducted using a Synergy H1 microplate reader (BioTek) at 543 nm excitation/584 nm emission. An area scan was performed from below using a 24‐well plate setting and a 7 × 7 matrix in each well. The average fluorescence intensity was calculated for the entire matrix. TAMRA experiments were repeated four times.

### 2.3 Rheology

A Kinexus Ultra+ rheometer (Malvern Instruments) was used to measure the storage moduli (G') of SAP gels. Gel volumes were 75 μl and were exposed to UV prior to being assembled in PBS unless otherwise described. A biopsy punch was used to cut and peel away the PET membrane from each insert so that the gel could be transferred to the rheometer stage without damage. Gels were measured at room temperature with a 1.8 mm gap between the upper 8 mm titanium plate and quartz lower plate. A frequency sweep between 0.1 and 10 Hz was performed with constant strain of 1%, followed by an amplitude sweep from 0.1 to 10% strain at 1 Hz. G' was calculated by averaging the measured values across the frequency sweep for which the moduli was generally frequency independent and inertial effects were not dominant. The mismatch between the area of the titanium plate and the gel was corrected for by multiplying moduli values by the ratio of plate area to gel area (~1.223). All measurements were repeated on 3–4 different days, with 2 gels measured per day yielding 6–8 replicates.

### 2.4 Atomic force microscopy

SAP solutions were made at 1 mg/mL in sterile water, exposed to UV if indicated, and then diluted to 0.1 mg/ml in sterile water and placed in a sonicator bath for 30 min. Next, 50 μl of each solution was deposited onto freshly cleaved mica and allowed to adsorb to the surface for 5 min. Excess liquid was blotted from the mica using filter paper, and samples were stored at 4°C overnight to finish drying. Images were obtained using a Bruker multimode AFM with NanoScope IIIa controller. The AFM probe had a resonance frequency of 325 kHz and a force constant of 40 N/m (MikroMasch #HQ:NSC15/AL BS). Scanning was performed in tapping mode with a 2 Hz scan rate and 512 samples/line. AFM height images were corrected for low‐frequency noise and tilt by flattening them with NanoScope Analysis software v1.5 (Bruker).

### 2.5 Cell culture and gel encapsulation

Human mesenchymal stem cells (MSC, Lonza #PT‐2501) were cultured in low‐glucose DMEM (Gibco #11054) containing 10% fetal bovine serum (FBS, Sigma #F4135), 1 ng/ml human fibroblast growth factor (PeproTech #100‐18B), 2 mM L‐glutamine (Gibco #25030), 50 U/ml penicillin and 50 U/ml streptomycin (Life Technologies #15140) and were used up to passage 20. HT1080 human fibrosarcoma cells were obtained from ATCC (#CCL‐121); cultured in RPMI 1640 (Gibco #21870) containing 10% FBS, 2 mM L‐glutamine, 50 U/ml penicillin and 50 U/ml streptomycin; and were used up to passage 20. For gel encapsulation experiments, cells were pelleted and resuspended in 20% (wt/vol) sucrose (Fisher Sci #S5500) in sterile distilled water and mixed 1:1 with SAP solutions. Due to the highly acidic nature of stock SAP solutions, 0.1 M NaOH (Fisher Sci #S318) dissolved in sterile distilled water was added to all SAP mixtures prior to mixing with cell solutions, for a final concentration of 4 mM NaOH unless otherwise stated.

### 2.6 Cell viability quantification

Cell viability was measured in 45 μl gels containing 3.34 mM KFE, 0.54 mM KFE‐alloc, 2 mM LAP, and 0.5 mM RGD, 4 mM NaOH, with or without 60 seconds of UV exposure. Gels containing encapsulated cells were assembled in culture media, and additional culture media was pipetted on top of gels after 25 min at 37°C and 5% CO_2_. Cells were also encapsulated in Matrigel (Corning, #354234) as a positive viability control, gelled at 37°C and 5% CO_2_ for 25 min, and then culture media was added to the well. After 24 h, a standard live/dead assay was performed according to the manufacturer's instructions (Invitrogen #L3224). A Nikon A1R live cell confocal microscope was used to take five random fields of view within each gel at 10x magnification using GFP and Texas Red channels. A custom CellProfiler software routine was written to quantify the number of live and dead cells in each image.^60^ Briefly, an adaptive threshold is calculated for each pixel using the “Robust Background” method, and cells at least 10 pixels in diameter are counted.

### 2.7 Optimization of NaOH neutralization

Cells were encapsulated in 75 μl gels for 24 h as previously described. A series of SAP solutions were pre‐neutralized so that gels would have a final NaOH concentrations ranging from 0 to 8 mM. After 24 h of incubation at 37°C and 5% CO_2_, alamarBlue (Invitrogen #DAL1025) cell viability reagent was added at a 1:10 dilution to the media surrounding each gel, according to manufacturer protocol. After 6 h of incubation, the fluorescent signal was measured using a plate reader at 560 nm excitation/590 nm emission. This extended incubation time was chosen based on previous studies.^59^, ^61^

### 2.8 Clicked‐in RGD effect on cell morphology

The cell adhesion peptide CRGDS, referred to here as “RGD,” was synthesized by Biomatik. Following gel assembly and UV exposure for 2D cell spreading experiments, gels were rinsed in PBS on a rocker overnight at 4°C. The next day, 1.5 × 10^4^ HT1080 cells/cm^2^ were seeded on top of the gels in standard culture media without FBS and cultured for 48 h at 37°C and 5% CO_2_. FBS‐free media was used so that cell adhesive proteins in serum did not adsorb to gel surface and cause spreading independent of RGD peptide. Adverse effects of serum starvation such as floating cells or apoptotic bodies were not observed. A custom CellProfiler routine was written to quantify the morphological characteristics of every cell. Briefly, a global minimum cross‐entropy thresholding method is used to identify individual cells, and the “MeasureObjectSizeShape” module is used to extract their morphological measurements. Over 300 cells per condition were quantified, across four replicate experiments.

For 3D cell encapsulation experiments, 2x concentration SAP solutions, partially neutralized with 4 mM NaOH, were mixed 1:1 with cells in 20% sucrose to obtain a final cell density of 2.5 × 10^5^ cells/ml. Gels were generated with a volume of 45 μl. Gels were exposed to UV as indicated and were allowed to assemble in MSC culture media for 25 min before adding additional media on top of gels. To demonstrate the ability to click a functionality into an already formed gel, the gels assembled without RGD or LAP were incubated in media supplemented with RGD and LAP for 1 h prior to any UV exposure. To explore the role of MMPs, in some cultures, GM6001 (Abcam #ab120845) was supplemented in cell culture media for a final concentration of 10 μM. Encapsulated cells were then cultured for 48 h at 37°C and 5% CO_2_.

To fluorescently stain actin, cells were fixed in 4% PFA for 30 minutes and rinsed with PBS. Cells were then permeabilized in 0.5% TritonX‐100 (Sigma #X100) in PBS for 5 min and simultaneously stained with phalloidin 488 (abcam # ab176753) and Hoechst 33342 (Invitrogen #H3570) for 1 h rocking at room temperature. Cells were rinsed in PBS three times for 10 min each, and five random fields of view were taken in each gel using a Nikon A1R live cell confocal microscope, with 10x magnification and 2x zoom in the DAPI and GFP channels. A custom Cellprofiler routine was used to detect individual cells in images and quantify their morphological characteristics.

### 2.9 Fluorogenic biosensor activation by MMP

The fluorogenic MMP biosensor Dabcyl‐GGPQG↓IWGQK‐Fluorescein‐AeeaC (where ↓ indicates the protease cleavage site) was synthesized using SPPS and functionalized with a quencher (dabcyl) and fluorophore (fluorescein) as previously described.^50^, ^62^ SAP hydrogels with the MMP biosensor clicked‐in were generated at a volume of 45 μl. For experiments testing exposure to collagenase, gels were incubated in various concentrations of collagenase dissolved in PBS (ThermoFisher cat#17100–017 at 100, 50, 20, 10, 5, 2.5, 1, 0.1, 0.01 μg/ml) and incubated for 24 h at 37°C and 5% CO_2_. For experiments involving cell encapsulation, gels were assembled and incubated in standard MSC media, but with 10% FBS replaced with 1% charcoal‐stripped serum, prepared in our lab as described previously,^59^ to reduce background biosensor activation by serum proteases. Following gel assembly and UV exposure, fluorescence measurements of the biosensor fluorescein signal within the hydrogels were conducted using a Synergy H1 microplate reader (BioTek) at 487 nm excitation/528 nm emission. An area scan was performed from below using a 24‐well plate setting and a 7 × 7 matrix in each well. The average fluorescence intensity was calculated for the entire matrix. Empty wells were filled with PBS to prevent evaporation.

### 2.10 Statistical analysis

Statistical analysis was performed using GraphPad Prism v9.3. Rheological data was fit using nonlinear regression and plotted with both axes on a log scale. The *p*‐value of this correlation was calculated by log‐transforming both variables and performing a linear regression. Data presented are the mean ± standard deviation (SD) unless otherwise noted. Differences in cell viability and morphological measurements between gels were analyzed using a one‐way ANOVA with Tukey's multiple comparison posttest. Differences in fluorescence signal versus control in MMP biosensor assays were analyzed using a one‐way ANOVA with Dunnett's multiple comparison posttest. Differences in retained TAMRA signal over time between gels was analyzed using a two‐way ANOVA with Tukey's multiple comparison posttest (both PBS washing time and UV exposure time were assessed as sources of variation). Statistical significance was defined using α = 0.05.

## 3 RESULTS

### 3.1 Thiol‐ene click chemistry binds fluorescent dye to alloc‐modified SAP hydrogel

**FIGURE 1:**
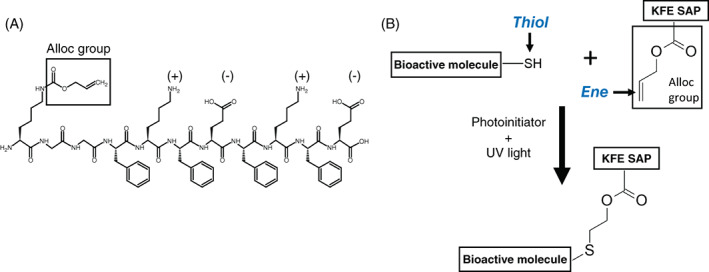
Diagram of KFE‐alloc structure and thiol‐ene reaction. (A) Chemical structure of KFE‐Alloc with side‐chain charges indicated. (B) Thiol‐ene click chemistry reaction forms a covalent bond between a thiolated bioactive molecule and KFE‐alloc SAP. SAP, self‐assembling peptide

**FIGURE 2:**
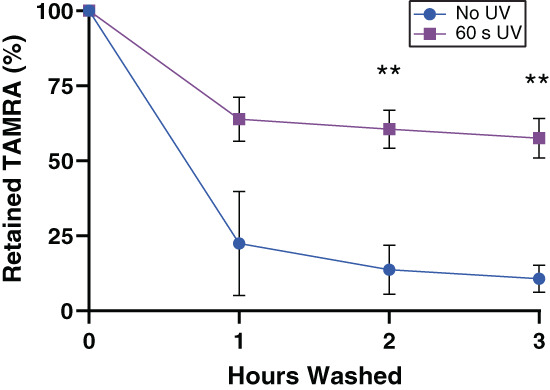
TAMRA dye clicked into SAP hydrogels using thiol‐ene chemistry. Thiol‐ene click chemistry (initiated by UV light) causes an increase in TAMRA retention in alloc‐modified SAP hydrogels compared to gels in which the reaction is not initiated. Mean ± SD, **p < .01 versus no UV control. SAP, self‐assembling peptide; TAMRA, 5(6)‐carboxytetramethylrhodamine

SAPs were modified with an alloc group to enable functionalization through an easy and cytocompatible thiol‐ene click chemistry reaction. KFE SAP was synthesized with a lysine‐alloc at the N terminus to enable thiol‐ene click chemistry reactions (Figure 1). This alloc provides the “ene” to which thiolated ligands are bound. To verify incorporation of molecules into SAP hydrogels using this chemistry, a cysteine amino acid (thiol) labeled with the fluorescent dye TAMRA was “clicked” into 3D SAP hydrogels—TAMRA‐C. The concentration of KFE‐alloc (0.54 mM) used throughout this work was chosen based on the amount of KFE‐RGD (KFE synthesized with an attached RGD group) used in previous works.^17^, ^40^ The TAMRA fluorescent signal retained in the gel was measured by a plate reader immediately after UV light exposure and over time after several PBS rinses (Figure 2). After 3 h of washing, gels exposed to UV retained approximately five times more TAMRA signal as compared to gels that were not exposed to UV, indicating a covalent click reaction between the TAMRA‐C and the alloc‐modified SAP. A reduction in the absolute amount of fluorescent signal at time zero between UV exposed gels and non‐UV exposed gels indicated possible photobleaching and/or photodamage to the TAMRA dye (Supp. Figure 1). Other durations of UV exposure did not yield significant differences in TAMRA retention (Supp. Figure 1), therefore 60 seconds was chosen for subsequent reaction studies to balance reaction efficacy with potential damage to cells and molecules caused by UV and free radicals. Low doses of low intensity, long wavelength UV light typically do not damage cells, and the radicals created through photoinitiation are consumed by the reaction, minimizing impact on cell health.^63^

### 3.2 Mechanical characterization of click‐modified SAP hydrogels

**FIGURE 3:**
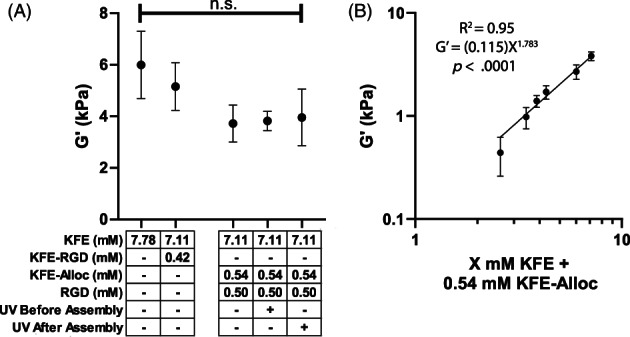
SAP mechanical properties are sensitive to peptide concentration but not RGD incorporation by click chemistry. (A) Incorporating RGD into Click SAPs via click chemistry does not significantly change the elastic moduli of the hydrogels, regardless of whether RGD is incorporated into the Click SAP before or after assembly. The densities of KFE‐RGD and KFE‐Alloc used are equivalent at 0.78 mg/ml. Mean ± SD, *N* = 3–4 experiments, 2 gels per experiment. No significant differences found. (B) Storage moduli of gels containing constant 0.54 mM KFE‐alloc and clicked‐in RGD increases with KFE concentration. SAP, self‐assembling peptide

Since substrate stiffness can affect cell behaviors such as migration,^64^ differentiation,^65^ and proliferation,^66^ and the viscosity of gels can impact cell spreading^67^ and the rate of endocytosis,^68^ we characterized the effect of clicking in molecules to the SAP hydrogels on the material properties of the gels. Previous studies demonstrated that incorporation of the cell adhesion peptide RGD into the base KFE peptide during the chemical synthesis of the peptide, rather than afterwards via click chemistry, reduced the stiffness of the resulting gel,^40^ with the magnitude of the decrease in stiffness dependent on the amount of RGD incorporated. Consistent with the published results, we observed a small (~14%, not statistically significant) decrease in stiffness between gels formed from the same total mass of KFE or KFE/KFE‐RGD mixture (Figure 3A, two left‐most columns). When measuring the effect of different SAP molecule modifications on overall gel stiffness (Figure 3A), we chose to have the same total mass of SAP in each gel so that total SAP density did not become a confounding variable in our comparisons. The storage moduli (G') of gels with RGD clicked in were compared to identical gels where the reaction was not initiated by UV, and no statistically significant differences were observed (Figure 3A, right columns, loss moduli G" and tanδ in Supp. Figure 2A,B). Clicking the RGD onto the SAPs before or after gel assembly both resulted in similar stiffnesses compared to gels without UV initiation, suggesting that click chemistry can be done without significantly altering mechanical properties. A non‐significant decrease in stiffness on average was observed in Click SAP gels compared to gels with the same total mass density of pure KFE, which agrees with previous rheological studies comparing modified and unmodified SAP gels.^40^

Gel stiffness could be tuned by varying the concentration of KFE while holding the concentration of KFE‐alloc constant. A range of substrate stiffnesses (~450–3800 Pa) was achieved (Figure 3B, loss moduli and tanδ in Supp. Figure 2C,D). This showed a strong correlation with a power law relationship (R^2^ = 0.95, Equation (1)) as seen previously in KFE SAP gels.^40^

(1) $$G^{\prime }=0.115{\left(C\right)}^{1.783}.$$

*G'* in kPa; *C* is [KFE] in mM.

### 3.3 AFM of the fibrous microarchitecture of SAP

**FIGURE 4:**
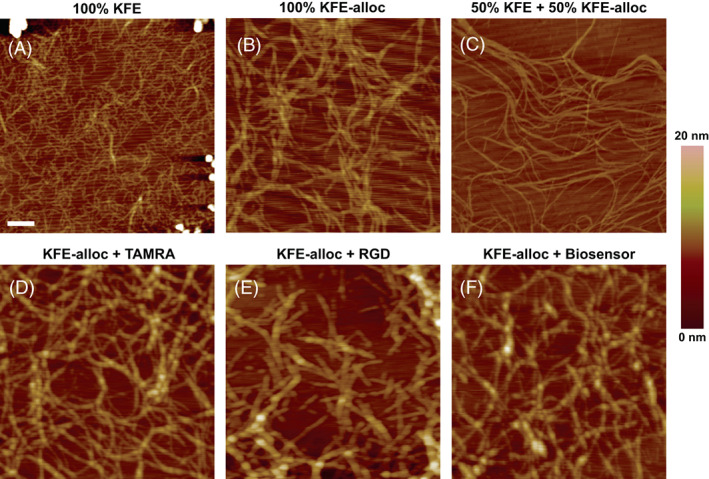
SAP modification does not interfere with fiber assembly. Atomic force microscopy images of 0.1 mg/ml aqueous solutions of (A) pure KFE, (B) pure KFE‐alloc, and (C) a 50/50 mixture of KFE and KFE‐alloc fibers show that modifying the KFE sequence with an alloc group does not inhibit the formation of fibers. Scale bar = 250 nm. Color bar represents the height of each pixel. Images taken after KFE‐alloc was then functionalized with (D) TAMRA, (E) RGD, and (F) QGIW biosensor peptide demonstrate that clicking in these molecules also does not interfere with the formation of a fibrous network. SAP, self‐assembling peptide; TAMRA, 5(6)‐carboxytetramethylrhodamine

KFE SAP have previously been shown to exhibit a fibrous structure when diluted and dissolved in water,^30^, ^37^ making it possible to assess this structure using AFM. Scans of 0.1 mg/ml solutions of SAP fibers deposited on cleaved mica demonstrate that alloc modification of either 100% or 50% of KFE peptides does not prevent fiber formation (Figure 4). Additionally, functionalizing the SAP fibers by clicking in TAMRA, RGD, or MMP biosensor did not prevent the SAPs from forming a fibrous network, which mimics the interconnected fibrous structure of stromal ECM, especially collagen, in vivo.

### 3.4 Cell spreading on top of SAP hydrogels with clicked‐in RGD

**FIGURE 5:**
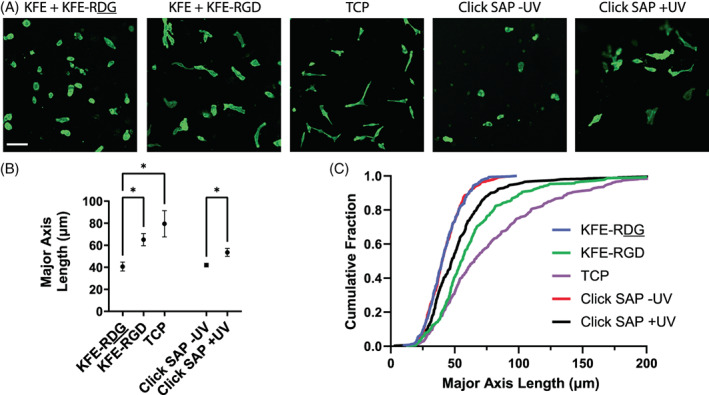
Incorporation of RGD into SAP gels via click chemistry promotes cell spreading in 2D. (A) Representative images of HT1080 fibrosarcoma cells cultured for 48 h in 2D on top of SAP gels containing non‐adhesive KFE‐RDG or adhesive KFE‐RGD, on TCP, or on gels containing RGD incorporated via thiol‐ene reaction (Click SAP) with and without UV initiation. Actin cytoskeleton labeled with fluorescent phalloidin. Scale bar = 100 μm. (B) Plot indicates the mean and standard deviation of the major axis length on each type of gel. *N* = 4 replicate experiments. *p < .05 via one‐way ANOVA with Tukey multiple comparisons post‐test. Not all significances shown. (C) Empirical cumulative distribution functions of the major axis lengths of all cells on each substrate across all four replicate experiments. SAP, self‐assembling peptide; TCP, tissue culture plastic; TAMRA, 5(6)‐carboxytetramethylrhodamine

The small peptide RGD, an integrin binding site derived from the ECM protein fibronectin, is often incorporated into biomaterials to promote cell attachment and survival.^69^ Here, we tested whether RGD incorporated into SAP hydrogels using thiol‐ene chemistry would retain biological activity and promote spreading of HT1080 cells on top of the gels (Figure 5). HT1080 fibrosarcoma cells are relevant for studying cancer cell migration, ECM remodeling, and drug treatment response. RGD click gels were constructed with 7.11 mM KFE, 0.54 mM KFE‐alloc, 2 mM LAP, and 0.5 mM RGD. One set of these gels was exposed to UV light for 60 s prior to washing, while another set was not exposed. KFE‐RGD and KFE‐RDG scrambled non‐adhesive control gels contained 7.04 mM KFE, and 0.54 mM of either KFE‐RGD or KFE‐RDG. Cells were also plated on TCP as a positive control for cell spreading. The major axis length, or the length of the major axis of an ellipse fit to the area of each cell, was quantified using CellProfiler software, with additional morphological metrics found in Supp. Figure 3. Consistent with previous reports,^40^ introducing an integrin binding peptide at the time of chemical synthesis of the peptide increased cell spreading, but the use of a scrambled version of the peptide (KFE‐RDG) did not (Figure 5). Similarly, RGD incorporated in the Click SAP gels increased the average spreading of cells on top of the gels (Figure 5B) and the percentage of cells exhibiting a high amount of spreading (Figure 5C) compared to Click SAP gels without UV initiation. Importantly, adding the RGD peptide to the KFE‐alloc mixture, with no exposure to UV light to initiate the thiol‐ene reaction, did not support cell spreading compared to the scrambled control, indicating that the adhesion peptide needed to be clicked into the SAP matrix to support cell adhesion on top of the gels.

### 3.5 Viability of cells encapsulated in 3D Click SAP gels

**FIGURE 6:**
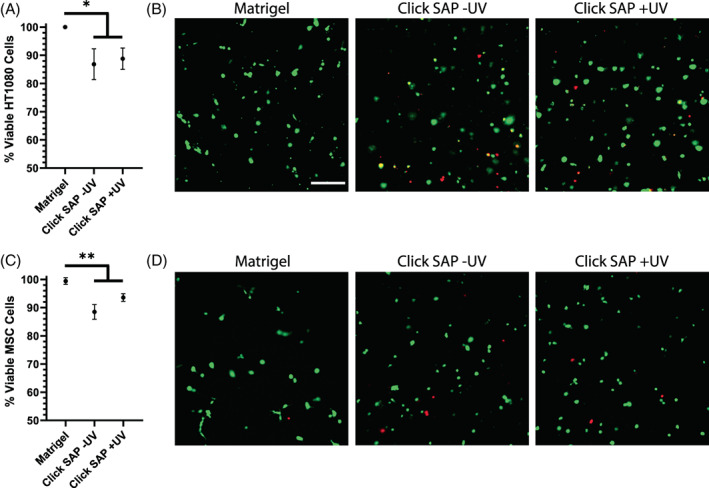
Multiple cell types remain highly viable during SAP encapsulation and click reaction. HT1080 cells (A and B) and MSCs (C and D) were encapsulated in SAP gels with RGD clicked in to measure the effects of the SAP, 60 seconds of UV light, and the click reaction on cell viability. Cells were also encapsulated in Matrigel as a control, along with SAPs that were not exposed to UV. 24 h after encapsulation and clicking in RGD, a live/dead assay was performed to quantify the number of live cells (green) and dead cells (red). A custom CellProfiler pipeline was written to quantify the overall percentage of live cells in each experiment (A, C). Points indicate the mean ± SD, *N* = 3–4 replicate experiments, *p < .05, **p < .01. Representative images demonstrate the effects of gel conditions on cell viability (B, D). Scale bar = 250 μm. MSC, mesenchymal stem cells; SAP, self‐assembling peptide

To develop Click SAP gels as a model system for physiologically relevant 3D cell culture, the viability of encapsulated HT1080s and MSCs was optimized. MSCs are a model mesenchymal cell line with relevance to wound healing, ECM production, and cancer. Aqueous solutions of unassembled SAP can be highly acidic^26^, ^70^ and thus were partially neutralized prior to mixing in cells. A final concentration of 4 mM NaOH was found to sufficiently neutralize the SAP solution to support cell viability during encapsulation, without triggering premature gel assembly (see Supp. Figure 4 for NaOH optimization). In initial viability experiments, 75 μl gels were used, but viability was generally at or below ~70%. We reasoned that generating thinner gels would decrease the time required for media to diffuse throughout the gel and fully neutralize the cells microenvironment, therefore improving viability. In subsequent experiments, the thinner 45 μl gels consistently had viability at or above ~85%. Once optimized, viability was quantified using a live/dead assay and compared to a Matrigel control, an established natural hydrogel for 3D cell culture (Figure 6). Additionally, the effect of the click reaction on viability was also tested. Culturing cells in Click SAP hydrogels caused a decrease in viability compared to matrigel, but overall viability remained relatively high. HT1080 cells were over 85% viable on average, and MSCs were over 88% viable, neither of which decreased with thiol‐ene reaction exposure. These results suggest that the cells did not experience prolonged free radical exposure during the click reaction. Thus, Click SAP gels and the thiol‐ene reaction itself are biocompatible and suitable for 3D cell encapsulation studies.

### 3.6 Spreading of cells encapsulated in 3D Click SAP gels

**FIGURE 7:**
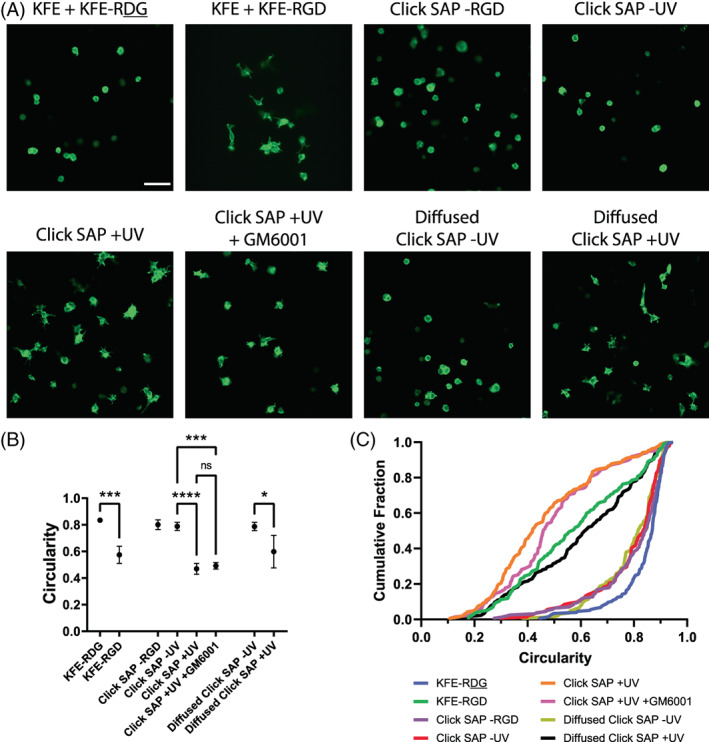
Incorporation of RGD into SAP gels via click chemistry promotes stem cell spreading in 3D. (A) Representative images of MSCs encapsulated for 48 h in 3D SAP gels show that clicking in RGD promotes cell spreading in a non‐MMP dependent manner. Actin cytoskeleton stained with fluorescent phalloidin. GM6001 is a general MMP inhibitor (10 μM). Scale bar = 100 μm. (B) Plots of the mean and standard deviations of the circularity of cells within each type of gel as measured with CellProfiler. *N* = 3 replicate experiments. *p < .05, ***p < .0005, ****p < .0001. Not all significances shown. (C) Empirical cumulative distribution functions of circularities for all cells across all replicate experiments. MSC, mesenchymal stem cells; SAP, self‐assembling peptide

We next determined whether clicked‐in RGD can promote cell adhesion and spreading for cells encapsulated in 3D, in addition to the 2D configuration. Spreading of MSCs in these gels was compared to control gels containing equivalent concentrations of KFE and either KFE‐RGD or KFE‐RDG, along with click gels containing no RGD (Figure 7A). Click SAP Gels contained final concentrations of 3.34 mM KFE, 0.54 mM KFE‐alloc, 2 mM LAP, and 0.5 mM RGD. KFE‐RGD and KFE‐RDG scrambled non‐adhesive control gels contained 3.34 mM KFE, and 0.54 mM of either KFE‐RGD or KFE‐RDG. CellProfiler software was used to quantify the circularity of each cell, which is defined as 4π*Area/Perimeter^2^. This metric equals 1 for a perfectly circular object and decreases toward zero as objects become less circular. This metric was found to capture the differences in cell spreading within 3D gels better than the major axis length previously used for cells on top of gels, likely because spreading within gels tends to be isotropic, rather than along one axis as seen in cells on gels. Additional morphological metrics can be found in Supp. Figure 5. As with 2D gels, cells show significantly more spreading with KFE‐RGD present compared to KFE‐RDG (Figure 7). Cells within click SAP gels with RGD covalently bonded to the matrix show significantly more spreading than gels not exposed to UV (i.e., when the RGD is present but not bound to the matrix). To determine whether this spreading was MMP‐dependent, a general MMP inhibitor GM6001 was added to the media, and the thiol‐ene RGD reaction was initiated. The GM6001 caused no significant change in cell circularity (*p* > .99), suggesting that the RGD‐dependent cell spreading is MMP‐independent.

Finally, to test the ability to temporally control presentation of biochemical cues in alloc‐modified SAPs, RGD and LAP were diffused into gels for 1 h after assembly, rather than being mixed into the initial SAP solution. In these gels, subsequent UV initiation of the thiol‐ene reaction also significantly increased cell spreading, demonstrating that biochemical cues can be added to the SAPs at desired time points following cell encapsulation. The distribution of MSC circularities in SAP gels with RGD clicked in was similar to that of gels containing KFE‐RGD, while those without RGD clicked in resembled that of gels containing scrambled KFE‐RDG (Figure 7C).

### 3.7 SAP hydrogels functionalized with a fluorogenic MMP biosensor

**FIGURE 8:**
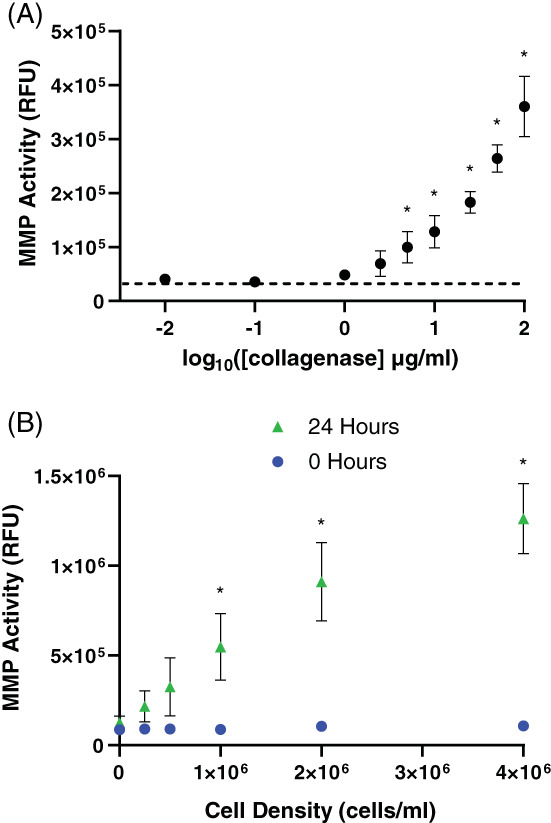
Degradable FRET‐based biosensor incorporated into SAP gels via click chemistry detects MMP activity. (A) Increase in the fluorescence intensity of the FRET‐based biosensor after incubating SAPs in increased concentrations of collagenase in PBS after 24 h. Enzymes degrade the QGIW sequence, which releases the fluorescein dye from the dabcyl quencher. Mean ± SD, *N* = 3 replicate experiments. *p < .05 versus PBS blank (dotted line). (B) Fluorescence intensity of the biosensor at 0 and 24 h with various seeding densities of MSCs. Mean ± SD, *N* = 3–4 replicate experiments. *p < .05 versus 0 cells/ml. SAP, self‐assembling peptide

Hydrogels have previously been functionalized with a FRET‐based peptide MMP sensor to measure MMP activity in situ. This has been performed in PEG hydrogels to study cell‐derived MMP activity^50^, ^61^ and for high‐throughput drug screening,^59^, ^71^ but has not previously been used to create 3D “smart” reporter SAPs. Here, thiol‐ene chemistry was used to bind this biosensor to the Click SAP matrix, and the sensitivity of the biosensor to exogenous collagenase (a mixture of MMPs isolated from bacteria), as well as cell‐secreted enzymes, was measured (Figure 8). Click SAP gels contained 3.16 mM KFE, 0.54 mM KFE‐alloc, and 0.25 mM of clicked‐in MMP biosensor, and were incubated for 24 h in collagenase, which resulted in a dose‐dependent increase in fluorescein signal as the peptide was cleaved and the fluorescein was no longer quenched by dabcyl (Figure 8A). Doses of 5 μg/ml collagenase (log_10_[5] ~ 0.7) and above caused significant increases in fluorescence signal compared to PBS‐only control. On average, 100 μg/ml collagenase caused a > 7‐fold increase in fluorescence signal over the time period.

MSCs were encapsulated in Click SAP gels containing final concentrations of 2.45 mM KFE, 1.08 mM KFE‐alloc, 0.5 mM clicked‐in RGD, and 0.25 mM clicked‐in MMP biosensor. After 24 h of culture, the fluorescent signal increased in a cell density‐dependent manner (Figure 8B). Cell densities of 1 × 10^6^ cells/ml and above caused significant increases in fluorescence signal compared to controls without cells. Of note, gels containing 4 × 10^6^ cells/ml exhibited a > 11‐fold change on average in fluorescent signal after 24 h.

## 4 DISCUSSION

In an effort to create a simple and flexible system to modify SAP, we used click chemistry to introduce functionality into the blank slate of KFE, and demonstrate that the resulting Click SAP retains the desirable mechanical and micro‐architectural properties of the original SAP. Click chemistry has been widely used in other biomaterials, and in addition to its ease of use, its potential benefits include spatial and temporal control of biomaterial properties,^54^, ^56^ and the ability to covalently link a wide range of molecule types and sizes.^47^ While a complete demonstration of the potential uses of the Click SAP system is beyond the scope of this initial report, we illustrated the flexibility and utility of the Click SAP by introducing three different classes of functionality: a fluorescent label, an integrin binding site, and an MMP‐sensitive biosensor.

Characterization of Click SAPs with AFM imaging indicates that the fibrous microarchitecture of the original KFE SAP is retained after introduction of the alloc group or clicking in the RGD containing integrin binding sequence (Figure 4). Retention of the microarchitecture is crucial since, as summarized previously, one of the most appealing features of SAP systems that distinguish them from most other synthetic hydrogels is that SAP have a fibrous microarchitecture similar to stromal ECM^16^ and the microarchitecture of a substrate is a key determinant of cellular responses to it.^5^ Careful inspection of AFM images, however, will reveal slight differences in the fiber dimensions between unmodified KFE and the various modified KFEs. This change in fiber dimensions is not an unexpected outcome and it is not specific to the Click SAP. The unmodified KFE was originally designed and chosen based on its structure that supports self‐assembly. It is reasonable to expect that inclusion of additional amino acids such as the integrin binding sequence could perturb this assembly. We^30^, ^40^ and earlier work by others^41^ report that adding integrin binding sites to a SAP during peptide synthesis results in fibers of altered dimensions as observed by AFM. Interestingly, though the addition of the alloc to the KFE resulted in a visible change in fiber dimensions, clicking in the fluorescent label, the integrin binding site, or the biosensor resulted in no obvious further changes in the fiber dimensions. Thus, at least for the three different functionalities we investigated, clicking a molecule in the SAP did not noticeably alter the fiber dimension relative to the KFE‐alloc, which is the natural control condition to use for experiments.

Rheology suggested that a range of physiologically relevant gel moduli could be achieved in Click SAPs (Figure 3). Taking an example from cancer biology, the elastic moduli of both normal (G′ ~ 0.1–0.4 kPa) as well as cancerous mammary tissue (G′ ~ 3–5 kPa) could be achieved with this Click SAP system.^72^, ^73^, ^74^ Our measurements followed a power law relationship between SAP concentration and elastic moduli as those previously reported for unmodified KFE.^40^ Addition of the alloc moiety to KFE reduced the stiffness of the resulting gels. As with the AFM observations of altered fiber dimension, this effect on stiffness is consistent with our previous work^30^, ^40^ and earlier work by others^41^ reporting that adding integrin binding sites to a SAP during peptide synthesis results in less stiff gels compared to the unmodified SAP. Clicking in the RGD peptide had no additional change in stiffness relative to the KFE‐alloc (*p* > .98). Taken together, both the AFM and rheology measurements suggest that, at least with the molecules investigated, clicking molecules into the KFE‐alloc has little impact on the microarchitecture or stiffness of the resulting gels, thus potentially allowing users to tune desired properties (e.g., integrin binding site density) without causing significantly confounding changes in gel physical properties. It should be noted that all the molecules we clicked into the gel were ~1–2 kDa, so future studies will be needed to investigate the effect of larger molecules (e.g., proteins) on the physical properties of SAP.

Stable incorporation of a fluorescent dye to the hydrogel demonstrated that thiol‐ene click chemistry could be used to covalently link a thiolated molecule to SAP fibers containing free alloc groups. After rinsing unreacted TAMRA out of the gel, it was found that the UV‐initiated reaction caused a significant (5x) increase in retained signal (Figure 2). While 60 s was chosen as an example UV duration in this study, this may need to be adjusted to account for changes in UV source intensity, gel dimensions, the amounts of thiols and enes in the solution, cell type, the sensitivity of constituent molecules to photodamage and photobleaching, and other factors. Because the thiol‐ene reaction is initiated with light, future applications could harness this feature using photomasks or multiphoton microscopy to perform 2D and 3D spatial patterning of biochemical signals within this system.^49^, ^54^, ^56^ Spatial patterning is not possible when biochemical signals are incorporated prior to gel assembly, but could be important for accurately mimicking aspects of native ECM, which is inherently dynamic, complex, and heterogeneous on many length scales.^75^ Additionally, we were able to control presentation of cell adhesion cues in time by clicking in RGD post‐self assembly (Figure 7). This ability to temporally control the properties of Click SAPs could be harnessed to model the microenvironment of complex, progressive diseases whose biochemical properties evolve over time. This is not possible in traditional SAPs, where functional moieties are added during the synthesis process or entrapped during hydrogel assembly. Mechanical properties could also be temporally controlled by introducing dithiol crosslinkers to stiffen gels on demand. For example, this temporal stiffening could be used to study how cells respond to a transition from a normal to cancerous physical microenvironment.^76^, ^77^

While others have used click chemistry to incorporate assembling peptides into PEG gels,^2^, ^3^, ^4^ the present study is the first to show that biologically active molecules can be clicked into a hydrogel comprised solely of SAP fibers. Previous functionalization of SAP fibers via thiol‐ene chemistry were not verified via cell interaction and were not performed in 3D hydrogels. To demonstrate this, we show that Click SAP with RGD covalently linked to the matrix promotes cell spreading in both 2D and 3D (Figures 5 and 7). RGD is a common integrin binding site and can be used to tune the cellular adhesiveness of a material.^69^ We found that cell spreading in 3D occurred even in the presence of a MMP inhibitor GM6001, suggesting that spreading was independent of MMP activity (Figure 7). This highlights a benefit of using ECM‐like fibrous biomaterials such as Click SAP to study cell behavior. In dense, nonfibrous nanoporous gels such as Matrigel or PEG, cells must use a proteolytic migration mode to degrade the surrounding matrix, while in a fibrous material, cells can switch to a protease‐independent amoeboid mechanism in order to migrate.^5^, ^78^ Thus, a fibrous material such as Click SAP could be used to investigate the role of MMPs in cell invasion beyond ECM degradation, such as in growth factor activation, induction of epithelial‐mesenchymal transition, or cleavage of cell surface adhesion receptors.^79^

The Click SAP platform can be used to generate “smart” SAP biomaterials by covalently incorporating biosensors such as an MMP‐sensitive fluorogenic peptide (Figure 8). This functionalized hydrogel could be used to study how 3D biophysical and biochemical cues, drugs, and other factors regulate cellular MMP activity in a fibrous tissue. In this study, the clicked‐in MMP biosensor was able to measure a range of collagenase concentrations, as well as the MMP activity of encapsulated cells, showing significant sensitivity to as little as 1 × 10^6^ MSCs per ml (45,000 total cells). Since the fluorescein‐labeled end of the biosensor remains covalently linked to the matrix, even after peptide degradation by MMPs, future studies could investigate the spatial location of MMP activity in real‐time using optical sectioning fluorescence microscopy techniques such as confocal microscopy^17^, ^30^ or multiphoton microscopy.^80^

Another advantage of Click SAPs over existing biomaterials used for disease modeling is affordability. The total cost of the constituents in the Click SAPs with RGD used to encapsulate cells (e.g., as in Figure 7) is approximately $10/ml, much less than the widely used Matrigel, which is typically $25–30/ml. Additionally, because peptides can be purchased from vendors, no specialized techniques or skills are required, and no special equipment is needed other than a UV lamp (~$500).

In summary, functionalized Click SAPs allow one to conveniently introduce various functionality in the blank slate of the KFE SAP system while retaining a fibrous microarchitecture similar to stromal ECM. The modifications we demonstrated allow one to control both the mechanical (e.g., stiffness) and biochemical cues (e.g., integrin binding cite density) that the 3D ECM biomimetic presents to encapsulated cells, while at the same time using the matrix to monitor cellular activity (e.g., MMP‐sensitive biosensor). Based on the modifications we have already demonstrated, one can envision researchers exploiting these and other modifications to generate ECM biomimetics with specific tunable properties. For example, this platform could be used to build a new system to study how binding site identity and/or density affects the activity of cell‐secreted MMPs. Such ECM biomimetics could serve as in vitro models that better replicate the in vivo microenvironment, which could be useful to study physiological and pathological conditions and for drug screening.

## 5 CONCLUSIONS

Addition of a lysine with an alloc group to the previously studied KFE SAP allows multiple types of molecules to be covalently linked to SAP hydrogels through click chemistry thereby circumventing limitations associated with incorporating moieties prior to gel formation via solid phase peptide synthesis. The subsequent clicking in of various functional molecules did not disrupt the fibrous microarchitecture of the SAP or the ability of the user to control the mechanical properties of the resulting hydrogel–both important regulators of cell behavior. The resulting Click SAPs support cell encapsulation with high viability and provide spatiotemporal control of functionalization with biomolecules and biosensors. This platform can be used to generate smart SAP hydrogels that contain an MMP‐sensitive fluorogenic peptide, which can be used to study how cellular MMP activity is regulated in fibrous tissue.

## CONFLICT OF INTEREST

The authors declare no conflicts of interest.

## Supporting information


**Appendix S1** Supporting Information

## Data Availability

The data that support the findings of this study are available from the corresponding authors upon reasonable request.

## References

[1] Tibbitt MW , Anseth KS . Hydrogels as extracellular matrix mimics for 3D cell culture. Biotechnol Bioeng. 2009;103(4):655‐663. 19472329 10.1002/bit.22361 PMC2997742

[2] Pérez CMR , Rank LA , Chmielewski J . Tuning the thermosensitive properties of hybrid collagen peptide–polymer hydrogels. Chem Commun. 2014;50(60):8174‐8176. 10.1039/c4cc03171g 24926620

[3] Stahl PJ , Romano NH , Wirtz D , Yu SM . PEG‐based hydrogels with collagen mimetic peptide‐mediated and tunable physical cross‐links. Biomacromolecules. 2010;11(9):2336‐2344. 20715762 10.1021/bm100465q PMC3006224

[4] Hilderbrand AM , Ford EM , Guo C , Sloppy JD , Kloxin AM . Hierarchically structured hydrogels utilizing multifunctional assembling peptides for 3D cell culture. Biomater Sci. 2020;8(5):1256‐1269. 31854388 10.1039/c9bm01894h PMC7439559

[5] Hogrebe NJ , Reinhardt JW , Gooch KJ . Biomaterial microarchitecture: a potent regulator of individual cell behavior and multicellular organization. J Biomed Mater Res A. 2017;105(2):640‐661. 27682265 10.1002/jbm.a.35914

[6] Ma X , Schickel ME , Stevenson MD , et al. Fibers in the extracellular matrix enable long‐range stress transmission between cells. Biophys J. 2013;104(7):1410‐1418. 23561517 10.1016/j.bpj.2013.02.017 PMC3617419

[7] Canty EG , Starborg T , Lu Y , et al. Actin filaments are required for fibripositor‐mediated collagen fibril alignment in tendon. J Biol Chem. 2006;281(50):38592‐38598. 17020878 10.1074/jbc.M607581200

[8] de Vries HJC , Enomoto DNH , van Marle J , van Zuijlen PPM , Mekkes JR , Bos JD . Dermal organization in scleroderma: the fast Fourier transform and the laser scatter method objectify fibrosis in nonlesional as well as lesional skin. Lab Invest. 2000;80(8):1281‐1289. 10950119 10.1038/labinvest.3780136

[9] Drifka CR , Loeffler AG , Mathewson K , et al. Highly aligned stromal collagen is a negative prognostic factor following pancreatic ductal adenocarcinoma resection. Oncotarget. 2016;7(46):76197‐76213. 27776346 10.18632/oncotarget.12772 PMC5342807

[10] Conklin MW , Eickhoff JC , Riching KM , et al. Aligned collagen is a prognostic signature for survival in human breast carcinoma. Am J Pathol. 2011;178(3):1221‐1232. 21356373 10.1016/j.ajpath.2010.11.076 PMC3070581

[11] McLeod C , Higgins J , Miroshnikova Y , Liu R , Garrett A , Sarang‐Sieminski AL . Microscopic matrix remodeling precedes endothelial morphological changes during capillary morphogenesis. J Biomech Eng. 2013;135(7):71002. 23722263 10.1115/1.4023984

[12] Provenzano PP , Eliceiri KW , Campbell JM , Inman DR , White JG , Keely PJ . Collagen reorganization at the tumor‐stromal interface facilitates local invasion. BMC Med. 2006;4(1):38. 17190588 10.1186/1741-7015-4-38 PMC1781458

[13] Han W , Chen S , Yuan W , et al. Oriented collagen fibers direct tumor cell intravasation. Proc Natl Acad Sci U S A. 2016;113(40):11208‐11213. 27663743 10.1073/pnas.1610347113 PMC5056065

[14] Reinhardt JW , Gooch KJ . Agent‐based modeling traction force mediated compaction of cell‐populated collagen gels using physically realistic fibril mechanics. J Biomech Eng. 2014;136(2):021024. 24317298 10.1115/1.4026179

[15] Reinhardt JW , Gooch KJ . An agent‐based discrete collagen fiber network model of dynamic traction force‐induced remodeling. J Biomech Eng. 2018;140(5):13. 10.1115/1.4037947 28975252

[16] Sieminski AL , Semino CE , Gong H , Kamm RD . Primary sequence of ionic self‐assembling peptide gels affects endothelial cell adhesion and capillary morphogenesis. J Biomed Mater Res A. 2008;87A(2):494‐504. 10.1002/jbm.a.31785 18186067

[17] Hogrebe NJ , Gooch KJ . Direct influence of culture dimensionality on human mesenchymal stem cell differentiation at various matrix stiffnesses using a fibrous self‐assembling peptide hydrogel. J Biomed Mater Res A. 2016;104(9):2356‐2368. 27163888 10.1002/jbm.a.35755

[18] Sieminski AL , Was AS , Kim G , Gong H , Kamm RD . The stiffness of three‐dimensional ionic self‐assembling peptide gels affects the extent of capillary‐like network formation. Cell Biochem Biophys. 2007;49(2):73‐83. 17906362 10.1007/s12013-007-0046-1

[19] Luo Z , Yue Y , Zhang Y , et al. Designer D‐form self‐assembling peptide nanofiber scaffolds for 3‐dimensional cell cultures. Biomaterials. 2013;34(21):4902‐4913. 23602368 10.1016/j.biomaterials.2013.03.081

[20] Ashworth JC , Thompson JL , James JR , et al. Peptide gels of fully‐defined composition and mechanics for probing cell‐cell and cell‐matrix interactions in vitro. Matrix Biol. 2020;85–86:15‐33. 10.1016/j.matbio.2019.06.009 PMC7610915 31295578

[21] Mi K , Wang G , Liu Z , Feng Z , Huang B , Zhao X . Influence of a self‐assembling peptide, RADA16, compared with collagen I and matrigel on the malignant phenotype of human breast‐cancer cells in 3D cultures and in vivo. Macromol Biosci. 2009;9(5):437‐443. 19165822 10.1002/mabi.200800262

[22] Sheikholeslam M , Wheeler SD , Duke KG , Marsden M , Pritzker M , Chen P . Peptide and peptide‐carbon nanotube hydrogels as scaffolds for tissue & 3D tumor engineering. Acta Biomater. 2018;69:107‐119. 29248638 10.1016/j.actbio.2017.12.012

[23] Yang Z , Zhao X . A 3D model of ovarian cancer cell lines on peptide nanofiber scaffold to explore the cell–scaffold interaction and chemotherapeutic resistance of anticancer drugs. Int J Nanomed. 2011;6:303‐310. 10.2147/IJN.S15279 PMC3044183 21383855

[24] Betriu N , Semino CE . Three‐dimensional tumor‐stroma co‐culture system development using self‐assembling peptide scaffolds. Afinidad. 2019;76(587):8.

[25] Huang H , Ding Y , Sun XS , Nguyen TA . Peptide hydrogelation and cell encapsulation for 3D culture of MCF‐7 breast cancer cells. PLOS One. 2013;8(3):e59482. 23527204 10.1371/journal.pone.0059482 PMC3603912

[26] Hainline KM , Gu F , Handley JF , et al. Self‐assembling peptide gels for 3D prostate cancer spheroid culture. Macromol Biosci. 2019;19(1):1800249. 10.1002/mabi.201800249 PMC6333502 30324687

[27] Ling PMT , Cheung SWH , Tay DKC , Ellis‐Behnke RG . Using self‐assembled nanomaterials to inhibit the formation of metastatic cancer stem cell colonies in vitro. Cell Transplant. 2011;20(1):127‐131. 20887677 10.3727/096368910X532783

[28] Shah RN , Shah NA , Del Rosario Lim MM , Hsieh C , Nuber G , Stupp SI . Supramolecular design of self‐assembling nanofibers for cartilage regeneration. Proc Natl Acad Sci. 2010;107(8):3293‐3298. 20133666 10.1073/pnas.0906501107 PMC2840471

[29] Cunha C , Panseri S , Villa O , Silva D , Gelain F . 3D culture of adult mouse neural stem cells within functionalized self‐assembling peptide scaffolds. Int J Nanomed. 2011;6:943‐955. 10.2147/IJN.S17292 PMC3124398 21720506

[30] Stevenson MD , Piristine H , Hogrebe NJ , et al. A self‐assembling peptide matrix used to control stiffness and binding site density supports the formation of microvascular networks in three dimensions. Acta Biomater. 2013;9(8):7651‐7661. 23603000 10.1016/j.actbio.2013.04.002 PMC4487911

[31] Miroshnikova YA , Jorgens DM , Spirio L , Auer M , Sarang‐Sieminski AL , Weaver VM . Engineering strategies to recapitulate epithelial morphogenesis within synthetic three‐dimensional extracellular matrix with tunable mechanical properties. Phys Biol. 2011;8(2):026013. 21441648 10.1088/1478-3975/8/2/026013 PMC3401181

[32] Giri S , Acikgöz A , Pathak P , et al. Three dimensional cultures of rat liver cells using a natural self‐assembling nanoscaffold in a clinically relevant bioreactor for bioartificial liver construction. J Cell Physiol. 2012;227(1):313‐327. 21437901 10.1002/jcp.22738

[33] Pugliese R , Marchini A , Saracino GAA , Zuckermann RN , Gelain F . Cross‐linked self‐assembling peptide scaffolds. Nano Res. 2018;11(1):586‐602.

[34] Silva D , Natalello A , Sanii B , et al. Synthesis and characterization of designed BMHP1‐derived self‐assembling peptides for tissue engineering applications. Nanoscale. 2012;5(2):704‐718. 23223865 10.1039/c2nr32656f

[35] Kopesky PW , Vanderploeg EJ , Kisiday JD , Frisbie DD , Sandy JD , Grodzinsky AJ . Controlled delivery of transforming growth factor β1 by self‐assembling peptide hydrogels induces chondrogenesis of bone marrow stromal cells and modulates Smad2/3 signaling. Tissue Eng Part A. 2011;17(1–2):83‐92. 20672992 10.1089/ten.tea.2010.0198 PMC3011906

[36] Wu EC , Zhang S , Hauser CAE . Self‐assembling peptides as cell‐interactive scaffolds. Adv Funct Mater. 2012;22(3):456‐468.

[37] Marini DM , Hwang W , Lauffenburger DA , Zhang S , Kamm RD . Left‐handed helical ribbon intermediates in the self‐assembly of a β‐sheet peptide. Nano Lett. 2002;2(4):295‐299.

[38] Bowerman CJ , Nilsson BL . Review: self‐assembly of amphipathic β‐sheet peptides: insights and applications. Pept Sci. 2012;98(3):169‐184. 10.1002/bip.22058 22782560

[39] Hwang W , Marini DM , Kamm RD , Zhang S . Supramolecular structure of helical ribbons self‐assembled from a β‐sheet peptide. J Chem Phys. 2003;118(1):389‐397.

[40] Hogrebe NJ , Reinhardt JW , Tram NK , et al. Independent control of matrix adhesiveness and stiffness within a 3D self‐assembling peptide hydrogel. Acta Biomater. 2018;70:110‐119. 29410241 10.1016/j.actbio.2018.01.031

[41] Genové E , Shen C , Zhang S , Semino CE . The effect of functionalized self‐assembling peptide scaffolds on human aortic endothelial cell function. Biomaterials. 2005;26(16):3341‐3351. 15603830 10.1016/j.biomaterials.2004.08.012

[42] Jung JP , Nagaraj AK , Fox EK , Rudra JS , Devgun JM , Collier JH . Co‐assembling peptides as defined matrices for endothelial cells. Biomaterials. 2009;30(12):2400‐2410. 19203790 10.1016/j.biomaterials.2009.01.033 PMC2677558

[43] Silva GA , Czeisler C , Niece KL , et al. Selective differentiation of neural progenitor cells by high‐epitope density nanofibers. Science. 2004;303(5662):1352‐1355. 14739465 10.1126/science.1093783

[44] Kumada Y , Zhang S . Significant type I and type III collagen production from human periodontal ligament fibroblasts in 3D peptide scaffolds without extra growth factors. PLOS One. 2010;5(4):e10305. 20421985 10.1371/journal.pone.0010305 PMC2858666

[45] Chau Y , Luo Y , Cheung ACY , et al. Incorporation of a matrix metalloproteinase‐sensitive substrate into self‐assembling peptides – a model for biofunctional scaffolds. Biomaterials. 2008;29(11):1713‐1719. 18192002 10.1016/j.biomaterials.2007.11.046

[46] Gelain F , Bottai D , Vescovi A , Zhang S . Designer self‐assembling peptide nanofiber scaffolds for adult mouse neural stem cell 3‐dimensional cultures. PLoS ONE. 2006;1:e119. 17205123 10.1371/journal.pone.0000119 PMC1762423

[47] Hoyle CE , Lowe AB , Bowman NC . Thiol‐click chemistry: a multifaceted toolbox for small molecule and polymer synthesis. Chem Soc Rev. 2010;39(4):1355‐1387. 20309491 10.1039/b901979k

[48] Fairbanks BD , Schwartz MP , Halevi AE , Nuttelman CR , Bowman CN , Anseth KS . A versatile synthetic extracellular matrix mimic via thiol‐norbornene photopolymerization. Adv Mater. 2009;21(48):5005‐5010. 25377720 10.1002/adma.200901808 PMC4226179

[49] Sawicki LA , Kloxin AM . Design of thiol–ene photoclick hydrogels using facile techniques for cell culture applications. Biomater Sci. 2014;2(11):1612‐1626. 25717375 10.1039/c4bm00187g PMC4324132

[50] Leight JL , Alge DL , Maier AJ , Anseth KS . Direct measurement of matrix metalloproteinase activity in 3D cellular microenvironments using a fluorogenic peptide substrate. Biomaterials. 2013;34(30):7344‐7352. 23830581 10.1016/j.biomaterials.2013.06.023 PMC4077845

[51] Kharkar PM , Kiick KL , Kloxin AM . Designing degradable hydrogels for orthogonal control of cell microenvironments. Chem Soc Rev. 2013;42(17):7335‐7372. 23609001 10.1039/c3cs60040h PMC3762890

[52] He X , Ma J , Jabbari E . Effect of grafting RGD and BMP‐2 protein‐derived peptides to a hydrogel substrate on osteogenic differentiation of marrow stromal cells. Langmuir. 2008;24(21):12508‐12516. 18837524 10.1021/la802447v

[53] DeForest CA , Anseth KS . Photoreversible patterning of biomolecules within click‐based hydrogels. Angew Chem Int Ed. 2012;51(8):1816‐1819. 10.1002/anie.201106463 PMC3430005 22162285

[54] Polizzotti BD , Fairbanks BD , Anseth KS . Three‐dimensional biochemical patterning of click‐based composite hydrogels via Thiolene Photopolymerization. Biomacromolecules. 2008;9(4):1084‐1087. 18351741 10.1021/bm7012636

[55] Grim JC , Marozas IA , Anseth KS . Thiol‐ene and photo‐cleavage chemistry for controlled presentation of biomolecules in hydrogels. J Control Release. 2015;219:95‐106. 26315818 10.1016/j.jconrel.2015.08.040 PMC4656112

[56] Gramlich WM , Kim IL , Burdick JA . Synthesis and orthogonal photopatterning of hyaluronic acid hydrogels with thiol‐norbornene chemistry. Biomaterials. 2013;34(38):9803‐9811. 24060422 10.1016/j.biomaterials.2013.08.089 PMC3830935

[57] Qin J , Sloppy JD , Kiick KL . Fine structural tuning of the assembly of ECM peptide conjugates via slight sequence modifications. Sci Adv. 2020;6(41):eabd3033. 33028534 10.1126/sciadv.abd3033 PMC7541060

[58] Mahmoud ZN , Gunnoo SB , Thomson AR , Fletcher JM , Woolfson DN . Bioorthogonal dual functionalization of self‐assembling peptide fibers. Biomaterials. 2011;32(15):3712‐3720. 21353303 10.1016/j.biomaterials.2010.12.002

[59] Fakhouri AS , Weist JL , Tomusko AR , Leight JL . High‐throughput three‐dimensional hydrogel cell encapsulation assay for measuring matrix metalloproteinase activity. Assay Drug Dev Technol. 2019;17(3):100‐115. 30958702 10.1089/adt.2018.877

[60] Carpenter AE , Jones TR , Lamprecht MR , et al. CellProfiler: image analysis software for identifying and quantifying cell phenotypes. Genome Biol. 2006;7(10):R100. 17076895 10.1186/gb-2006-7-10-r100 PMC1794559

[61] Leight JL , Tokuda EY , Jones CE , Lin AJ , Anseth KS . Multifunctional bioscaffolds for 3D culture of melanoma cells reveal increased MMP activity and migration with BRAF kinase inhibition. Proc Natl Acad Sci. 2015;112(17):5366‐5371. 25870264 10.1073/pnas.1505662112 PMC4418884

[62] Deshmukh AA , Weist JL , Leight JL . Detection of proteolytic activity by covalent tethering of fluorogenic substrates in zymogram gels. Biotechniques. 2018;64(5):203‐210. 29793363 10.2144/btn-2018-0005

[63] McCall JD , Anseth KS . Thiol–ene photopolymerizations provide a facile method to encapsulate proteins and maintain their bioactivity. Biomacromolecules. 2012;13(8):2410‐2417. 22741550 10.1021/bm300671s PMC3421966

[64] Lo C‐M , Wang H‐B , Dembo M , Wang Y . Cell movement is guided by the rigidity of the substrate. Biophys J. 2000;79(1):144‐152. 10866943 10.1016/S0006-3495(00)76279-5 PMC1300921

[65] Murphy WL , McDevitt TC , Engler AJ . Materials as stem cell regulators. Nat Mater. 2014;13(6):547‐557. 24845994 10.1038/nmat3937 PMC4163547

[66] Hadjipanayi E , Mudera V , Brown RA . Close dependence of fibroblast proliferation on collagen scaffold matrix stiffness. J Tissue Eng Regen Med. 2009;3(2):77‐84. 19051218 10.1002/term.136

[67] Gong Z , Szczesny SE , Caliari SR , et al. Matching material and cellular timescales maximizes cell spreading on viscoelastic substrates. Proc Natl Acad Sci. 2018;115(12):E2686‐E2695. 29507238 10.1073/pnas.1716620115 PMC5866566

[68] Edwards DA , Gooch KJ , Zhang I , McKinley GH , Langer R . The nucleation of receptor‐mediated endocytosis. Proc Natl Acad Sci. 1996;93(5):1786‐1791. 8700836 10.1073/pnas.93.5.1786 PMC39859

[69] Hersel U , Dahmen C , Kessler H . RGD modified polymers: biomaterials for stimulated cell adhesion and beyond. Biomaterials. 2003;24(24):4385‐4415. 12922151 10.1016/s0142-9612(03)00343-0

[70] Caplan MR , Moore PN , Zhang S , Kamm RD , Lauffenburger DA . Self‐assembly of a β‐sheet protein governed by relief of electrostatic repulsion relative to van der Waals attraction. Biomacromolecules. 2000;1(4):627‐631. 11710192 10.1021/bm005586w

[71] Fakhouri AS , Leight JL . Measuring global cellular matrix metalloproteinase and metabolic activity in 3D hydrogels. Jove J Vis Exp. 2019;143:e59123. 10.3791/59123 30735180

[72] Singh A , Brito I , Lammerding J . Beyond tissue stiffness and bioadhesivity: advanced biomaterials to model tumor microenvironments and drug resistance. Trends Cancer. 2018;4(4):281‐291. 29606313 10.1016/j.trecan.2018.01.008 PMC5884450

[73] Butcher DT , Alliston T , Weaver VM . A tense situation: forcing tumour progression. Nat Rev Cancer. 2009;9(2):108‐122. 19165226 10.1038/nrc2544 PMC2649117

[74] Paszek MJ , Zahir N , Johnson KR , et al. Tensional homeostasis and the malignant phenotype. Cancer Cell. 2005;8(3):241‐254. 16169468 10.1016/j.ccr.2005.08.010

[75] Khetan S , Burdick JA . Patterning hydrogels in three dimensions towards controlling cellular interactions. Soft Matter. 2011;7(3):830‐838.

[76] Wiley KL , Sutherland BP , Ogunnaike BA , Kloxin AM . Rational design of hydrogel networks with dynamic mechanical properties to mimic matrix remodeling. Adv Healthcare Mater. 2022;11(7):2101947. 10.1002/adhm.202101947 PMC8986572 34936227

[77] Günay KA , Ceccato TL , Silver JS , et al. PEG–anthracene hydrogels as an on‐demand stiffening matrix to study Mechanobiology. Angew Chem Int Ed. 2019;58(29):9912‐9916. 10.1002/anie.201901989 PMC6660351 31119851

[78] Wolf K , Mazo I , Leung H , et al. Compensation mechanism in tumor cell migration: mesenchymal–amoeboid transition after blocking of pericellular proteolysis. J Cell Biol. 2003;160(2):267‐277. 12527751 10.1083/jcb.200209006 PMC2172637

[79] Kessenbrock K , Plaks V , Werb Z . Matrix metalloproteinases: regulators of the tumor microenvironment. Cell. 2010;141(1):52‐67. 20371345 10.1016/j.cell.2010.03.015 PMC2862057

[80] Jayawarna V , Ali M , Jowitt TA , et al. Nanostructured hydrogels for three‐dimensional cell culture through self‐assembly of fluorenylmethoxycarbonyl–dipeptides. Adv Mater. 2006;18(5):611‐614.

