# Supplementary material for: Click chemistry functionalization of self‐assembling peptide hydrogels
Source: J Biomed Mater Res A. 2022 Oct 10;111(3):389–403. doi: 10.1002/jbm.a.37460 (PMC10092743; doi:10.1002/jbm.a.37460)

**Supplemental Figures**

Supp. Table 1. Complete statistical analysis of cell morphology metrics

Supp. Fig. 1. TAMRA dye clicked into SAP hydrogels using thiol-ene chemistry with various amounts of UV light

Supp. Fig. 2. Additional mechanical properties of SAPs

Supp. Fig. 3. Additional morphological measurements of cells cultured in 2D on SAP gels

Supp. Fig. 4. NaOH optimization

Supp. Fig. 5. Additional morphological measurements of cells encapsulated in SAP gels


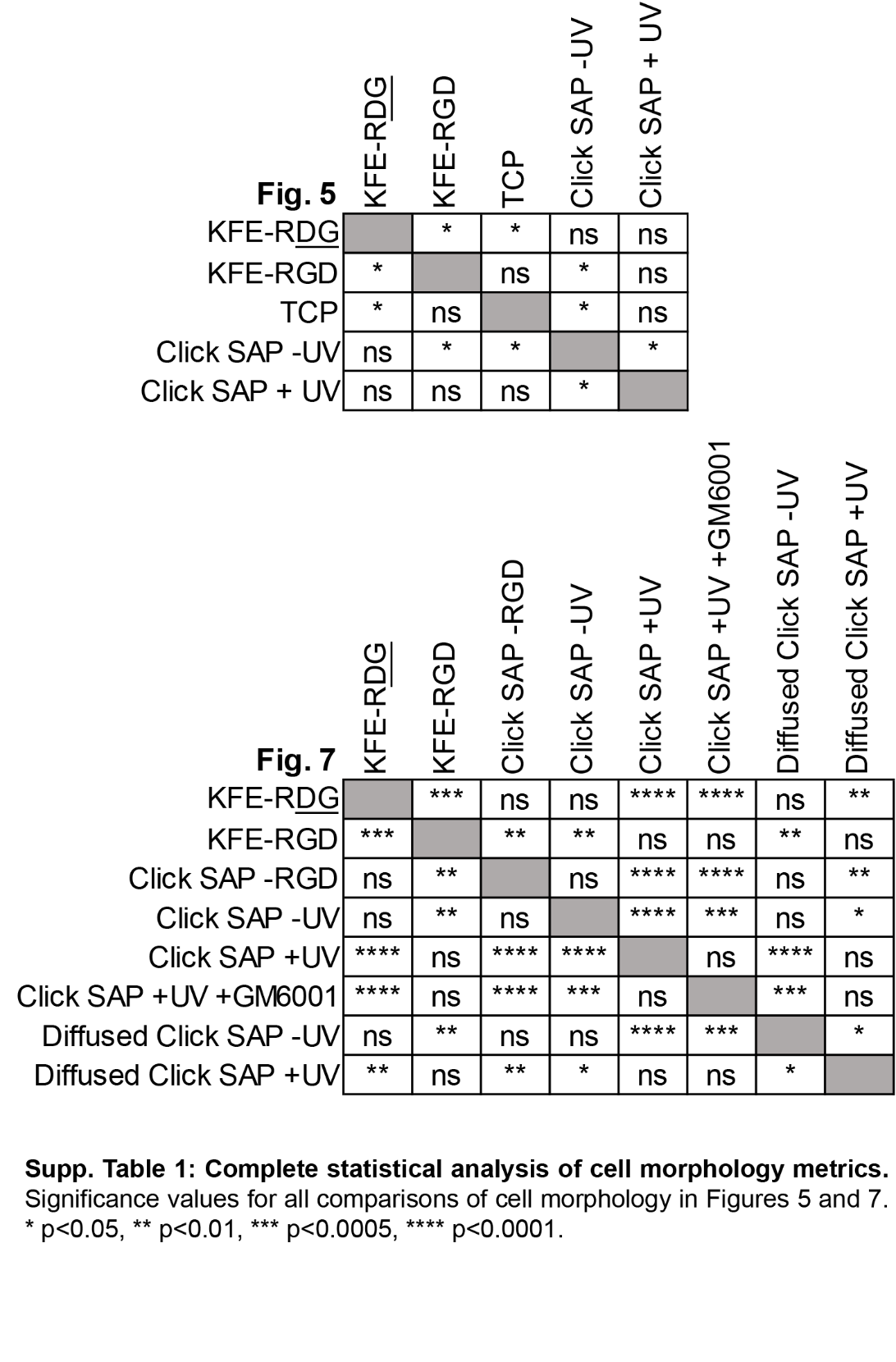


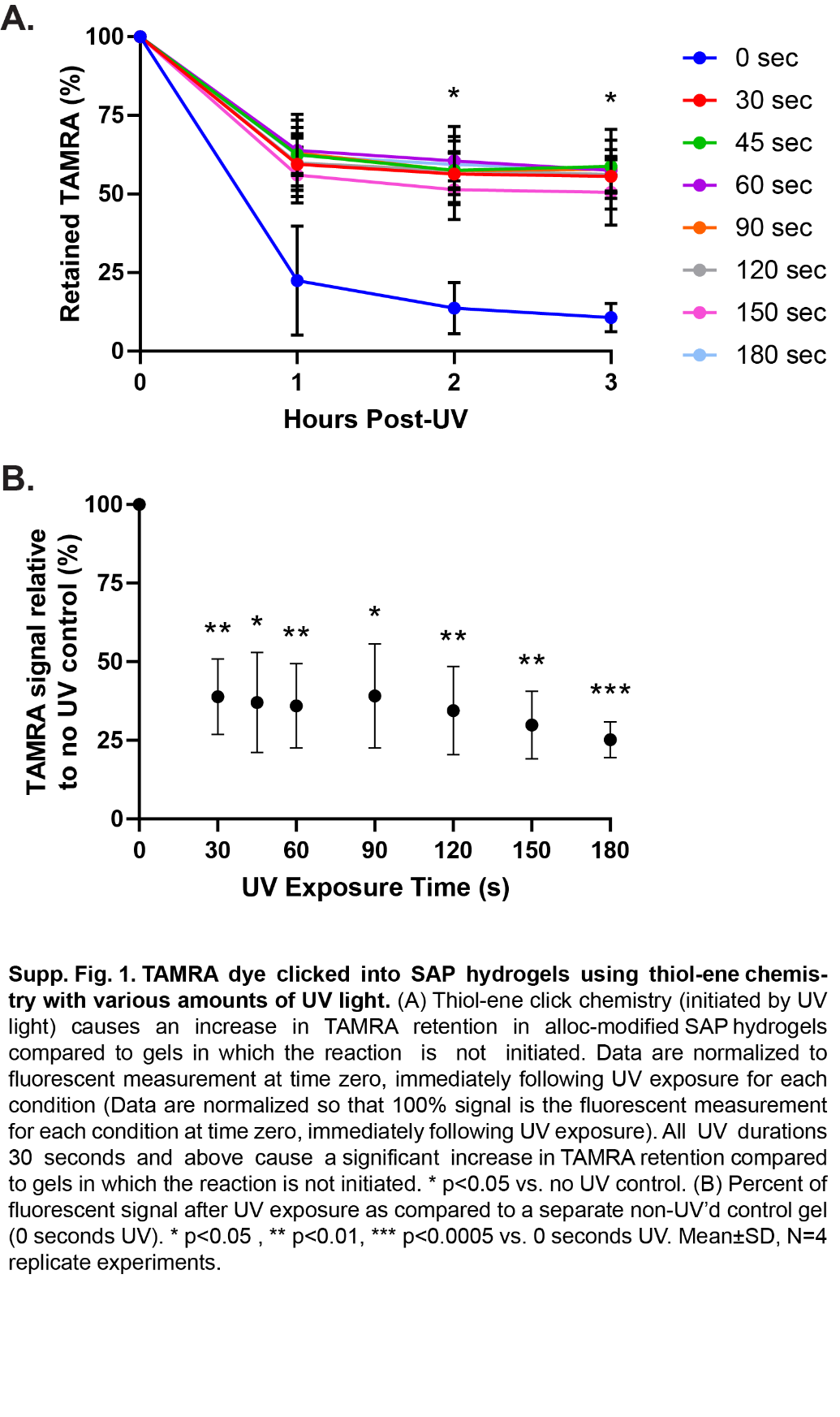


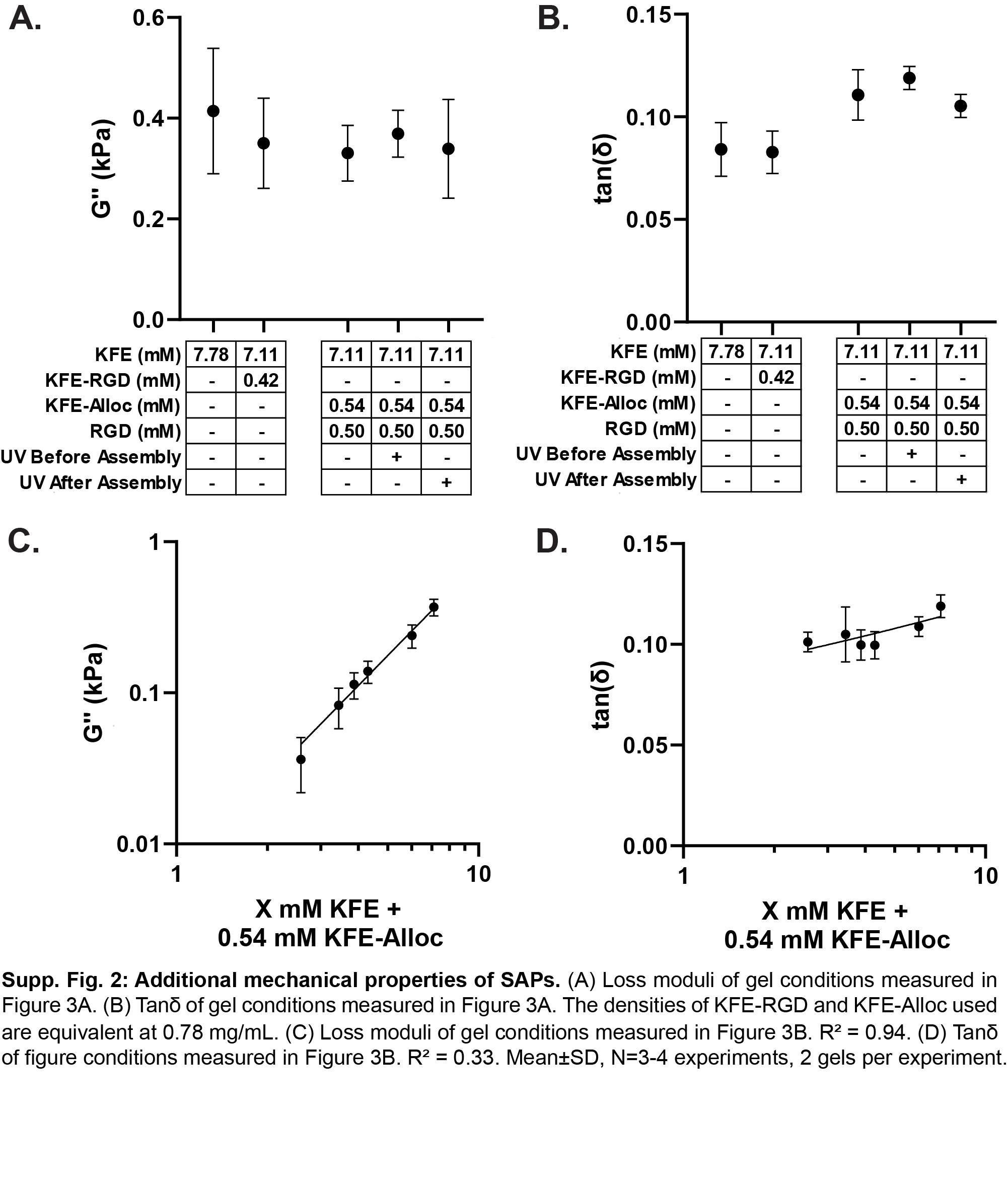


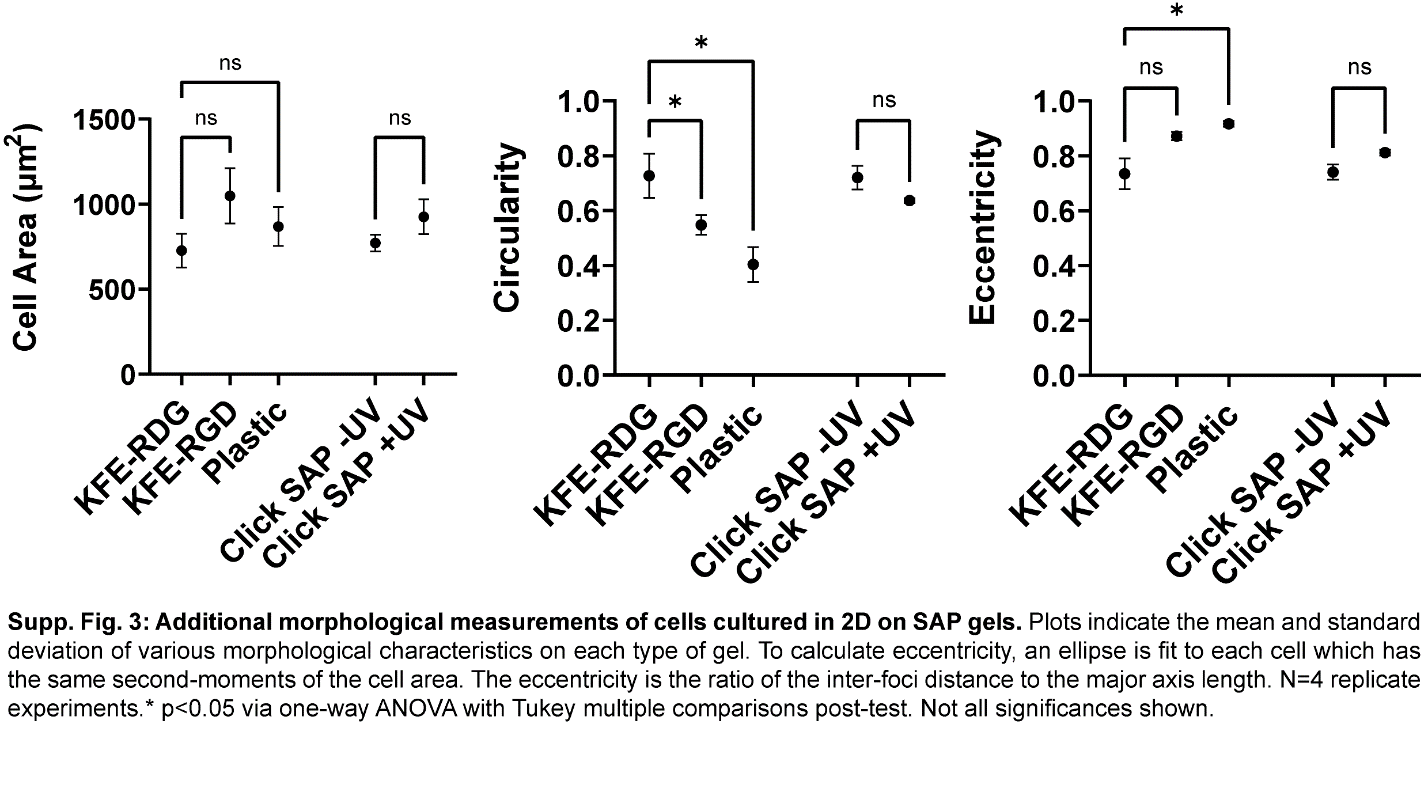

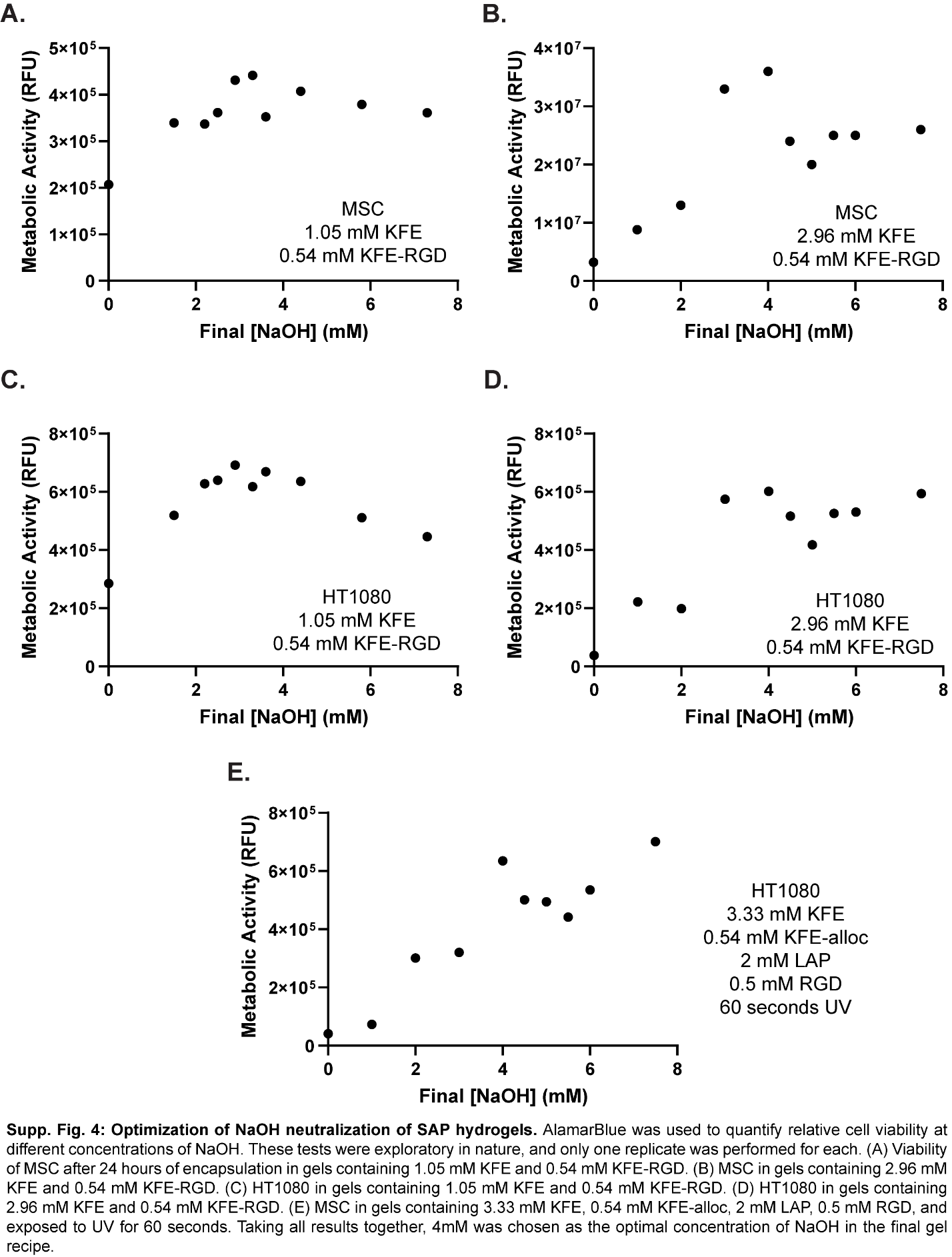


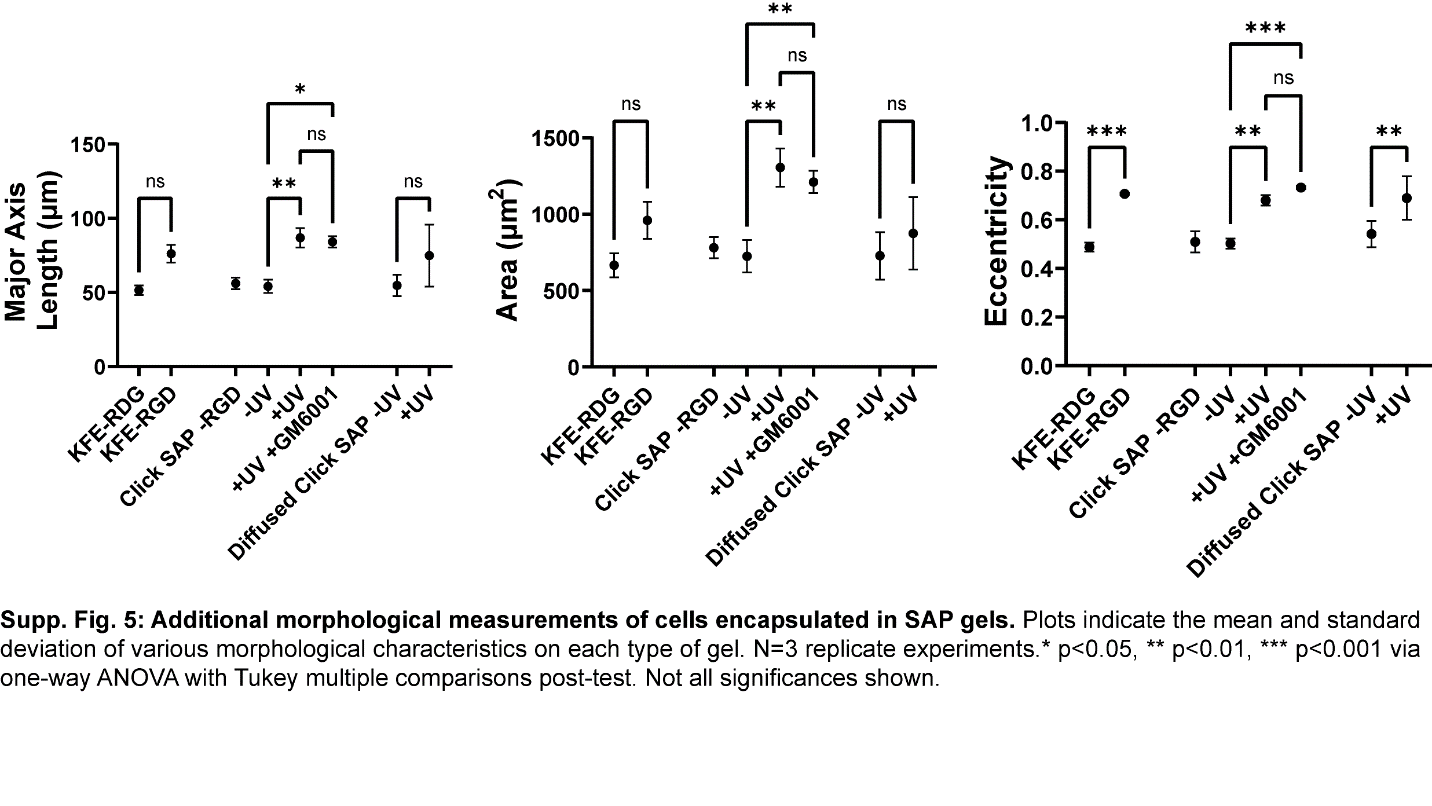

Supplement: Supplementary file 1 — Appendix S1 Supporting Information [file JBM-111-389-s001.docx]
